# Influence of clinical factors on the protective or deleterious impact of genetic variants in orthodontically induced external root resorption: an observational study

**DOI:** 10.1186/s12903-022-02306-y

**Published:** 2022-07-04

**Authors:** Henriqueta Coimbra Silva, Nuno Lavado, Filomena Canova, Miguel Guevara Lopez, Fernando J. Regateiro, Sónia A. Pereira

**Affiliations:** 1 Coimbra Institute for Clinical and Biomedical Research (iCBR), Faculty of Medicine, University of Coimbra, Coimbra, Portugal; 2 Institute of Medical Genetics/UCGenomics, Faculty of Medicine, University of Coimbra, Pólo 3. Subunidade 1, 3º andar, gab 77. Azinhaga de Sta Comba, Celas, 3000-548 Coimbra, Portugal; 3 Polytechnic of Coimbra, Institute of Engineering of Coimbra — ISEC, Coimbra, Portugal; 4 Department of Computing and Systems, Setúbal School of Technology, Polytechnic Institute of Setúbal, Campus de Estefanilha, Setúbal, Portugal; 5 Institute of Orthodontics, Faculty of Medicine, University of Coimbra, Coimbra, Portugal

**Keywords:** Complex traits, External apical root resorption, IL1RN, Orthodontics, P2RX7

## Abstract

**Background:**

Prediction of susceptibility to Orthodontically Induced External Apical Root Resorption (OIEARR) has been hampered by the complex architecture of this multifactorial phenotype. The aim of this study was to analyze the impact of the interaction of multiple variables in the susceptibility to OIEARR.

**Methods:**

The study evaluated 195 patients requiring orthodontic treatment. Nine clinical and treatment variables, single nucleotide polymorphisms (SNPs) from five genes and variables interactions were analyzed as risk factors for OIEARR using a multiple linear regression model.

**Results:**

The model explained 29% of OIEARR variability (ANOVA: *p* < 0.01). Duration of treatment was the most important predictor and gender was the second, closely followed by premolar extraction. For genes encoding osteoprotegerin (OPG), the receptor activator of nuclear factor κ B (RANK) and the IL1 receptor antagonist (IL1RN), the effect of analyzed variants changed from protective to deleterious depending on the duration of treatment and the age of the patient.

**Conclusions:**

This work shows that in OIEARR the impact of genetic susceptibility factors is dynamic changing according to clinical variables.

## Introduction

Accurate phenotype prediction is essential for precision medicine. Unfortunately, for complex diseases this task has not been well succeeded. Apart from the difficulty in characterizing the genetic component of inter-individual variability, the complex interaction between genetic and non-genetic variables is a major challenge.

In the field of orthodontic practice, orthodontically induced external apical root resorption (OIEARR) is a worrying iatrogenic effect. Approximately 30% of orthodontic patients suffer from moderate OIEARR, and in 2–5% the root loss is severe and threatens tooth stability [1, 2]. Many genetic and clinical variants have been explored to identify high risk patients. In orthodontic treatment, the success of tooth movement depends on the balance between applied forces and alveolar bone adaptive response [3, 4]. Biomechanical risk factors have been described as contributing to 10–30% of the variation observed in root resorption [5, 6]. The duration of treatment, type of appliance and tooth extraction have all been implicated [7, 8]. Some variants are not easily measured, such as the magnitude of force or intrusive movements, root torque, or extent of tooth movement [9, 10]. For alveolar bone modelling and remodeling, local release of inflammatory mediators, particularly of the interleukin 1 (IL1) pathway, stimulation of mechanotransduction receptors and interference with the differentiation of osteogenic (osteoblast) and osteolytic (osteoclasts) cells, are believed to play important roles [11, 12]. Genes encoding proteins involved in these pathways have been the target of multiple association studies [7, 13–15]. Although sib-pair [16, 17] and twin studies have pointed to a heritability of OIEARR between 50 and 84%, so far, no consensus exists on any analyzed genetic variant. Genetic variants with more consistent results are localized in loci involved in the interleukin 1 (IL1) pathway, like the IL1 beta (*IL1B) gene*, [13, 18, 19] the gene encoding the IL1 receptor antagonist, *IL1RN* [13] or the IL1 receptor-associated kinase1 (*IRAK1*) gene [20] . The gene encoding the purinergic receptor P2RX7, involved in mechanotransduction pathways [21] and genes encoding proteins with a prominent role in bone cells’ differentiation, like the receptor activator of nuclear factor-κB (RANK) or osteoprotegerin (OPG) [17, 22], have also been associated with susceptibility to OIEARR.

For OIEARR the analysis of variables interaction have been poorly explored and their dynamic nature has not been described. The aim of this study was to analyze the impact of variables interplay on an OIEARR prediction model.

## Materials and methods

### Study subjects

The study was performed according to the ethical principles governing medical research and human subjects as laid down in the Helsinki Declaration (2002 version, www.wma.net/e/policy/b3.htm). All patients were informed of all procedures and signed a written informed consent. The study was approved by the Ethics Committee of The Faculty of Medicine of the University of Coimbra.

Patients followed by the same orthodontist were randomly selected from the archives of two orthodontic clinics and from the Department of Orthodontics, Dentistry, Faculty of Medicine. The criteria used for patient selection were the following: complete comprehensive orthodontic treatment (straight-wire technique), existence of high-quality panoramic radiographs before and after treatment, unrelated and of Caucasian origin, with completely formed and erupted maxillary incisors and canines, with no craniofacial malformation and congenitally missing, supernumerary or impacted maxillary canines or incisors, as well as no aberrant morphology of roots that could interfere with length measurement. Patients that developed fractures, abrasion, or dental cavities on the incisal edges between measurements were excluded. The orthodontic treatment included the conventional straight-wire multibrackets, an appointment every month for the adjustment, the use of intraoral elastics and a final retention with the Hawley appliance in the upper arch and a fixed bonded metallic retention in the lower arch. When performed, pre-molar extraction included both upper first premolars and the use of stainless steel Goshgarian transpalatal bars to control anchorage.

From the review of 790 clinical records, 400 patients were selected according to these criteria. Of the 212 patients who agreed to participate, 17 were excluded, two for incomplete genetic data and 15 due to high influence (Cook’s D higher than 4/212 = 0.019) or outlier behavior (standardized residual values higher than 2 standard deviation (sd)), and 195 remained for further analysis [23].

Six maxillary teeth were assessed: the four incisors and the two canines. Patients’ characteristics, including variables that may influence OIEARR, are described in Table 1. For the sample size, with 12 variables and considering an individual effect size of 0.057, according to previous results, [21] a statistical power of 0.9 (for α = 0.05) is predicted, calculated using G*Power version 3 [24].

**Table 1:** Clinical characterization of patients

Characteristics	N (%)	%OIEARRmax 95%CI
Gender*		
Female	123 (63.1)	14.6–17.6
Male	72 (36.9)	18.4–23.3
Age (years)		
< 14	63 (32.3)	14.7–18.6
14–18	85 (43.6)	16.2–20.7
> 18	47 (24.1)	15.6–21.4
Months of treatment* (Mean 35.6; s.d.10.1)		
< 30 (1st tertile)	57 (29.2)	12.1–16.0
30–39	68 (34.9)	14.8–19.2
> 39 (2nd tertile)	70 (35.9)	19.5–24.3
Overjet (mm) (Mean 4.2; s.d.5.4)		
< 2 mm	43 (22.1)	16.7–23.1
2–3 mm	63 (32.3)	14.4–18.6
> 3 mm	89 (45.6)	15.9–19.9
Anterior open bite		
With	28 (14.4)	18.1–25.9
Without	167 (85.6)	15.8–18.6
Premolar extraction*		
With	58 (29.7)	19.0–23.6
Without	137 (70.3)	14.8–18.0
Tongue thrust		
With	58 (29.7)	16.4–21.5
Without	137 (70.3)	15.9–19.0
Angle classification [59]		
Class I	99(50.8)	14.9–18.5
Class II	79(40.5)	16.8–21.1
Class III	17(8.7)	14.1–25.8

^*^Significant difference between categories (p < 0.05)

*CI* Confidence Interval; *sd* standard deviation

### X-ray measurements

The classification of patients’ skeletal pattern was based on pre-treatment lateral cephalometric radiographs. Both panoramic radiographs (before and after treatment) were performed with the same equipment with the standard quality criteria of a panoramic X-ray. Panoramic radiographs were digitalized (300 dpi, 256 grey levels) using a scanner (Expression 1680 Pro, Epson) and saved in TIFF. The evaluation of OIEARR was performed by measuring root length variation in pre- and post-treatment panoramic radiographs using a specific software prototype (ARIAS for Apical Resorption Image Analysis System) as previously described [25]. A correction factor (CF) corresponding to the ratio between the initial (C1) and final crown lengths (C2), was applied, as it is accepted that during orthodontic treatment, the crown length does not change [25]. The applied formula was based on the Linge & Linge method [5] (1991) modified by Brezniak et al. [26] (2004) and calculates the percentage of root length variation (%OIEARR).

$$\% OIEARR = 1 - \left( {R1/R2*CF} \right)*100$$

where R1 and R2 are the initial and final root length, respectively, and CF = C1/C2

To better assess %OIEARR in each patient, the maximum value of %OIEARR obtained in the six teeth, %OIEARRmax, was considered as a dependent variable. All measurement procedures were executed by the same operator, a specialist in orthodontics.

### Genotyping

Five single nucleotide polymorphisms (SNPs) were analyzed (Table 2). DNA was extracted from buccal swabs using Chelex 100® (Sigma Aldrich, SL, USA). SNPs were identified as previously described: restriction fragment length polymorphism (RFLP) assays for rs1143634 (in *IL1B gene*) and rs3102735 (*TNFRSF11B gene*, encoding OPG) and TaqMan assays for other SNPs (rs315952 from *IL1RN gene*, rs1805034 from *TNFRSF11A gene*, encoding RANK, and rs1718119 from *P2RX7 gene*) [20, 21]. Some samples were genotyped by Sanger sequencing (primers sequence available on request) and used as positive controls in RFLPs and TaqMan assays. For simplicity, OPG and RANK will be used instead of HUGO Gene Nomenclature Committee (*HGNC*)-approved gene symbol.

**Table 2:** Frequencies of genotypes for analyzed SNPs

*RANK*		*OPG*		*IL-IB*		*P2RX7*		*IL1RN*	
Gen	N (Freq)	Gen	N (Freq)	Gen	N (Freq)	Gen	N (Freq)	Gen	N (Freq)
TT	79 (0.41)	TT	147 (0.75)	CC	104 (0.53)	GG	84 (0.43)	TT	110 (0.56)
CT	86 (0.44)	CT	43 (0.22)	CT	75 (0.39)	AG	89 (0.45)	CT	71 (0.37)
CC	30 (0.15)	CC	5 (0.03)	TT	16 (0.08)	AA	22 (0.11)	CC	14 (0.07)

*Gen* genotype; *N* number of patients; *Freq* frequency; *SNPs* single nucleotide polymorphisms

### Statistics

For intraoperator error analysis, the initial and final radiographs of 20 patients were randomly selected and evaluated twice by the same author, at an interval of 15 days. The differences in X-ray measurements were assessed by Student’s t-test for paired samples (systematic error) and by the Dahlberg formula (random error). Bland–Altman plots were also constructed for all six teeth measurements and intervals and 95% limits of agreement were calculated [27].

%OIEARRmax was evaluated as a quantitative variable using a multiple linear regression approach [23]. The values of %OIEARRmax were analyzed and compared between groups of patients divided according to clinical and treatment variables, using Student’s t-test and Mann–Whitney test [23] (Table 1). To keep statistical accuracy, as only 14 patients have used the Hyrax appliance, this variable was excluded from analysis. To assure statistical power, homozygous genotypes with fewer than thirty patients were pooled with heterozygous genotypes (Table 2). To identify risk factors associated with %OIEARRmax, a model using clinical, treatment and genetic variables, as well as their interactions, as predictors, was used. The selection of independent variables to be included in the model as predictors was based on previous results [21]. A screen of all two-way interactions with at least thirty individuals per group was performed. Interactions were visually explored using interaction plots and validated using linear models [23]. Model’s performance was estimated using adjusted R^2^ and the validation set approach. Using hold-out data (tenfold cross-validation with 10 runs), this approach allows us to estimate how well models will predict the phenotype in new patients and is an estimate for the test set RMSE (root mean squared error, denoted by CV-RMSE in the results section). The impact of variables on the model was evaluated using the absolute value of the *t*-statistic for each model parameter scaled to have a maximum value of 100 and minimum of 0. The model’s goodness of fit was assessed by residual analysis. The absence of multicollinearity and singularity was verified by examining tolerance and the variance inflation factor (VIF) [23]. The Hardy–Weinberg equilibrium was tested by contingency table comparisons of observed *vs.* expected genotypes’ frequencies. A *p* value of less than 0.05 was defined as statistically significant. The software environment R *version 3.4.3* [28] was used for statistical computing with the R library for classification and regression training, “caret” version 6.0–7 [29]. The datasets generated and analyzed during the current study are available in the Zenodo repository, [http://doi.org/10.5281/zenodo.1324556] [30].

## Results

**Fig. 1:**
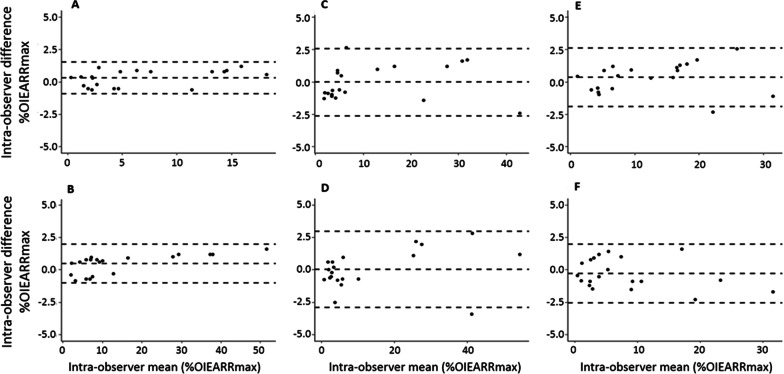
Bland–Altman plots for: **A** Tooth 13; **B** Tooth 12; **C** Tooth 11; **D** Tooth 21; **E** Tooth 22; **F** Tooth 23. The central line indicates the mean intra-observer difference and the upper and lower lines the 95% confidence interval limits

Intraoperator error analysis revealed that for systematic error (t-test) there were no statistically significant differences between the two measurements (*p* > 0.05); for random error (Dahlberg formula), values varied between 0.5% and 1%, which is not statistically significant [27]. This is also suggested by the Bland–Altman plots, as shown in Fig. 1, where teeth 13, 12, 11 and 23 all have points falling in the 95% confidence interval depicted by the upper and lower line, and teeth 21 and 22 have only 1 out of 20 data points falling outside the interval [27].

Clinical characterization of the patient sample is described in Table 1. The mean value of %OIEARRmax was 18.0% (sd 9.5%) for the total sample, 16.1% (sd 8.4%) for females, 20.9% (sd 10.6%) for males, 21.3% (sd 8.9%) for the 58 patients with premolar extraction and 16.4% (sd 9.4%) for the other 137 patients without premolar extraction. There was a statistically significant difference in %OIEARRmax between females and males, between patients with and without premolar extraction and between categories of treatment duration (*p* < 0.05). Data on OIEARR and %OIEARRmax distribution by tooth has already been described in previous work [25]. Frequencies of genotypes of the analyzed SNPs are shown in Table 2. The Hardy–Weinberg equilibrium was confirmed for all SNPs (*p* > 0.05).

**Fig. 2:**
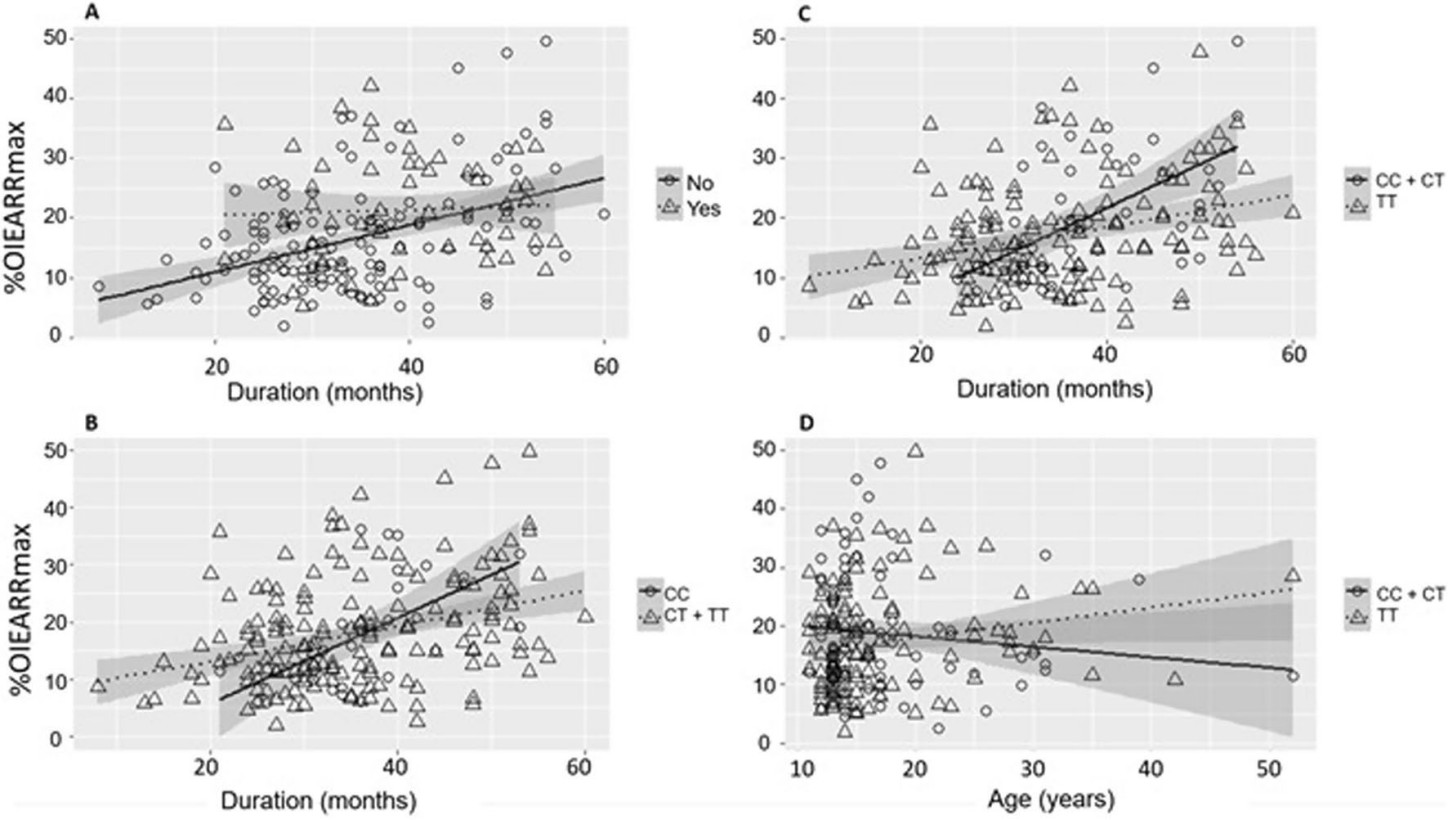
Interaction plots: **A** Duration versus premolar extraction; **B** Duration versus *RANK* genotype; **C** Duration vs. *OPG* genotype; **D** Age versus *IL1RN* genotype. Regression lines and their 95% confidence intervals are also displayed

Four “interactions” were identified by interaction plots and afterwards used in the model (Fig. 2): A) duration of treatment and premolar extraction (*p* < 0.001); B) duration of treatment and RANK gene SNP (*p* < 0.001); C) duration of treatment and OPG gene SNP (*p* < 0.001); D) patient age at the beginning of treatment and *IL1RN* genotype (*p* < 0.01). Besides these interactions, others have been tested but not used for further analysis (*p* > 0.05).

The regression model explains about 29% of %OIEARRmax variability (ANOVA: *p* < 0.01, n = 195, residual standard error = 8.00 on 182 degrees of freedom (df), adjusted R-squared = 0.29, coefficient of variance of the root mean square error (CV-RMSE) = 8.25). Table 3 describes the contribution of each variable and “interactions” in the model. Duration of treatment is the most important predictor of OIEARR and gender is the second, closely followed by premolar extraction and the interaction between duration of treatment and OPG gene SNP. Other genetic variants and “interactions” have lesser impact. After applying the False Negative Rate (FDR) correction for multiple testing, all the first nine variables of Table 3 remained statistically significant (p < 0.05) [31].

**Table 3:** Results of regression (N = 195)

	B	LCI	UCI	*p value*
(Intercept)	− 22.75	− 41.55	− 3.95	0.016
Duration of treatment (months)	1.09	0.6	1.58	< 0.001
Male	3.93	1.47	6.38	0.002
Premolar extraction	15.86	4.77	26.94	0.005
Duration:*OPG*, TT genotype	− 0.45	− 0.77	− 0.13	0.006
*OPG*, TT genotype (reference CC + CT genotype)	15.58	3.17	28.0	0.013
*P2RX7*, GG genotype (reference AA + AG genotype)	2.87	0.49	5.26	0.017
Duration: Premolar extraction	− 0.34	− 0.63	− 0.06	0.017
*IL1RN*, TT genotype (reference CC + CT genotype)	− 7.31	− 13.71	− 0.90	0.024
Age:*IL1RN*, TT genotype	0.38	0.03	0.73	0.031
Duration:*RANK*, CT + TT genotype	− 0.37	− 0.78	0.04	0.075
*RANK*, CT + TT genotype (reference CC genotype)	13.29	− 1.95	28.53	0.083
Age	− 0.07	− 0.34	0.20	0.590

*B* unstandardized coefficient; *LCI/UCI* 95% lower/upper confidence interval limit*.* Age was kept in the model due to its interaction with *IL1RN* SNP. Results are displayed from the most down to the least significant

If all other predictors remain constant, the model suggests that male patients have, on average, 4 percentage point (*p.p.)* (B = 3.93) more %OIEARRmax than females and that patients with genotype GG of *P2RX7* SNP have, on average, 3*p.p.* (B = 2.87) more %OIEARRmax (Table 3). For other contributing factors, the effects of interaction must be considered.

Based on non-standardized coefficients from Table 3 and in weighted averages by sample sizes within each subgroup, we could predict the effect of the interaction of “duration of treatment” and “age” with other variables. Patients submitted to premolar extraction have, on average, an overall 16*p.p.* (B = 15.86) more OIEARR, but are less affected by duration of treatment (2*p.p.* increase per year) than patients without premolar extraction (5*p.p.* increase per year). Therefore, for treatments up to 48 months, patients without premolar extraction remain less prone to OIEARR. Concerning the SNP of OPG gene, duration of treatment has less impact for patients with genotype TT than for patients with other genotypes (increase of 3*p.p. vs*. 8*p.p.* per year), so that for treatments up to 36 months TT is the risk genotype but for longer treatments it becomes protective. Similarly, genotypes CT and TT of RANK gene SNP are associated with a slower increase in OIEARR over time (3*p.p. vs*. 8*p.p.* per year) and though for treatments up to 36 months these are higher risk genotypes they become protective thereafter. Considering all the effects of interaction associated with the duration of treatment, the average effect of this variable is estimated to be 4*p.p.* per year. Analyzing the interaction between the “age at the beginning of treatment” and *IL1RN* SNP shows that after age 20, but not before, genotype TT becomes an increasing powerful risk factor (OIEARR increase of 0.31 *p.p.* for each additional year of age). Regression lines on Fig. 2A–C also suggested these effects.

## Discussion

In this work we explored not only clinical and genetic factors but also variables interaction effects determining susceptibility to OIEARR. Identified variables were used in a multiple linear regression model. The model explained 29% of inter-individual OIEARR variability, with an estimate for the test set RMSE (root mean squared error) of 8.25% OIEARR and a training set RMSE of 8% OIEARR, suggesting no overfitting of data.

Significant clinical contributors included duration of treatment, gender, age, and premolar extraction, all of them previously described in the literature, though with inconsistent results [8, 25, 32, 33]. The effect of gender is intriguing and may be related to differences in hormone profile influencing bone metabolism and mechanical stress. This susceptibility factor remains in the multiple linear regression model and is not explained by differences in other variables between females and males. From the 5 genes evaluated, three, *P2RX7,* OPG encoding gene and *IL1RN,* independently contributed to OIEARR susceptibility, and RANK encoding gene showed a near significant result.

The most interesting finding is the evidence of complex and dynamic variables interactions involving factors such as duration of treatment, premolar extraction, genetic profile, and age of patient. The harmful effect of long treatment duration has been described by many authors [6, 8, 20, 25] and may account for about 10% of OIEARR variation [7]. According to study results, the impact of treatment duration varies with patients’ characteristics, namely with the presence of premolar extraction, which is a powerful risk factor. Patients without premolar extraction have a lower risk of OIEARR, though in this group treatment duration is a critical factor. Otherwise, in the presence of premolar extraction, duration of treatment is less determinant. The impact of premolar extraction on OIEARR was reported by some [10, 34] but not all studies [6] and is probably related to the increased distance that canines and incisors must be moved in these patients, exposing their roots to a higher mechanical stress. The results also highlight an interaction between the duration of treatment, premolar extraction, and genetic profiles of OPG and RANK encoding genes. These proteins are involved in osteoclast differentiation, and it is interesting to observe the similarities in their interaction plot profiles (Fig. 2C, D). For the *IL1RN* TT genotype, the interference with susceptibility to OIEARR varies with patient age, changing from a protective to a risk factor.

According to the model, for the average 17-year-old patient, without premolar extraction, for a 30-month course of treatment, these results predict that higher risk patients will be male, with a genotype profile of GG for *P2RX7,* TT for OPG gene*,* TT or CT for RANK gene and CT or CC for *IL1RN*. For these patients, OIEARR will vary between 17 and 23% (95%CI). Results also predict that lower risk patients, females with the opposite genotypes, will have between 3 and 13% of OIEARR (95%CI). So, higher risk patients have 2.5 times more OIEARR than lower risk patients. For other clinical profiles, higher and lower risk genotypes may be different.

These types of cross-over interactions involving genetic and non-genetic variables have been described in complex phenotypes [35, 36]. In fact, the architecture of multifactorial phenotypes is multidimensional, and at least for some variables, when one changes, the impact of others will also change, with the pattern of interaction evolving over time [36]. Unfortunately, capturing this complexity is almost impossible and sample size, though, crucial, is probably not the main obstacle [35].

In a case/control study including sixty-seven patients and matched controls, Sharab et al. (2015) [7], who were also analyzing genetic and non-genetic variables, were able to explain about 25.13% of the variation in susceptibility to OIEARR. In previous work, the authors were able to explain 27% of this phenotype variability [20], and now, with 12 variables and exploring variable interaction, a slight improvement was achieved, but not enough to be clinically relevant. For clinical purposes, the low predictive power of these models discourages genotyping for personalization of treatment, but they still reveal critical factors that may have an impact on clinical management.

This study has some limitations. As revealed by the results of the multiple regression model, the size of the effect of variables is lower than predicted based on previous literature reports, meaning that a much larger sample size should be used in future work. Using %OIEARRmax as a dependent variable instead of data of all teeth improves the probability of identifying factors contributing to the phenotype, which is our main goal, but may overestimate the effects they have. Also, the exploratory approach used to select the two-way interactions to be included in the model may have introduced additional sources of noise. The method used for OIEARR diagnosis was based on panoramic films, known to be less accurate than periapical films. However, even periapical films show distortion because of error caused by variability in tooth shape and tooth angulation, and the literature review shows that independently of the methodology used, studies analyzing OIEARR etiology, severity, and distribution, give the same results [13, 15, 18]. Applying restricted technical quality criteria improves the accuracy for linear measurements on two panoramic radiographs [37]. Also, limitations of panoramic measurements are much less critical for maxillary teeth [38] and were even more reduced by the method implemented for measurements. Factors such as type of tooth movement and the magnitude of orthodontic forces were not evaluated because their accurate assessment in a clinical setting is still an insurmountable challenge.

In summary, this work highlights the complex architecture of OIEARR and shows for the first time that the effect of variables and their interactions is dynamic and evolves over time. Currently, genetic testing cannot be recommended to predict this iatrogenic event. The impact of unmodifiable variables like genetic profile, seems to be dependent on clinically manageable factors such as the age at which treatment is started and the duration of treatment, thus stressing the importance of judicious clinical decisions.

## Data Availability

The datasets generated and analysed during the current study are available in the Zenodo repository, [Data set]. Zenodo. http://doi.org/10.5281/zenodo.1324556.

## Abbreviations

OIEARR: Orthodontically Induced External Apical Root Resorption
P2RX7: Purinergic receptor P2X7
OPG: Osteoprotegerin
*IL1*: Interleukin 1
*IL1B*: Interleukin 1B
*IL1RN*: IL1 receptor antagonist
*IRAK1*: IL1 receptor-associated kinase1
RANK: Receptor activator of nuclear factor-κB
SNP: Single nucleotide polymorphism
RFLP: Restriction fragment length polymorphism
*TNFRSF11B*: Tumour Necrosis Factor Receptor Superfamily Member 11b gene, encoding osteoprotegerin (OPG)
*TNFRSF11A*: Tumour Necrosis Factor Receptor Superfamily Member 11a *gene*, encoding receptor activator of nuclear factor-κB RANK
